# Preemptive antibiotic strategies for gram-positive bacteria in preservation fluid: a single-center experience

**DOI:** 10.3389/fmed.2025.1665151

**Published:** 2025-09-10

**Authors:** Jianming Li, Jianyi Li, Jun Li, Qian Fu, Chenglin Wu, Huanxi Zhang, Xiaojun Su, Longshan Liu, Changxi Wang

**Affiliations:** ^1^Organ Transplant Center, First Affiliated Hospital, Sun Yat-sen University, Guangzhou, China; ^2^Department of Urology, The First Affiliated Hospital of Shandong First Medical University and Shandong Provincial Qianfoshan Hospital, Jinan, Shandong, China

**Keywords:** preemptive-anti-gram-positive antibiotic therapy, preservation fluid, infection, gram-positive bacteria, kidney transplant

## Abstract

**Objective:**

To elucidate the risk stratification of gram-positive bacteria in the preservation fluid (*PF*), investigate antibiotic resistance and its role in early post-kidney transplant infections, and assess the efficacy of preemptive-anti-Gram-positive antibiotic (P-antiGP) therapy.

**Methods:**

This retrospective study analyzed the clinical data of 144 kidney transplant donors and 218 recipients between April 2015 and October 2020. Recipients with any of the high-virulence gram-positive bacteria (such as *Enterococcus faecium*, *Enterococcus faecalis*, and *Staphylococcus aureus*) in *PF* were defined as high-risk group. Recipients with other pathogens in *PF* were defined as low-risk group.

**Results:**

The high-risk group had a significantly higher incidence of infection events as compared with the low-risk group (42.6% vs. 26.2%, *p* = 0.014). Multivariate analysis indicated a trend toward an increased risk of early post-transplant infections in the high-risk group (adjusted OR = 1.855, 95% CI: 0.991–3.464, *p* = 0.052). Seven recipients (1.5%) were diagnosed as possible donor-derived infections (P-DDIs) and all of them were from the high-risk group. 56.4% (123/218) of recipients had multidrug-resistant organisms (MDROs) in *PF* and 12.4% (27/218) had extensively-resistant organisms (XDROs). The P-DDIs rate was notably higher in the extensively drug-resistant (XDR) group than non-XDR group (11.1% vs. 2.1%, *p* = 0.014). The incidence of P-DDIs was significantly lower (*p* = 0.025) in recipients with P-antiGP therapy (4.3%) as compared to recipients who did not (23.8%).

**Conclusion:**

*E. faecium*, *E. faecalis*, and *S. aureus* in *PF* are considered high-virulence gram-positive bacteria, and recipients with these pathogens are categorized as high-risk group. Additionally, a high prevalence of antibiotic resistance exists among gram-positive bacteria in *PF*, correlating with post-transplant infections. Furthermore, The addition of P-antiGP therapy as a preemptive therapy in the high-risk group can effectively reduce the incidence of P-DDIs.

## Introduction

Kidney transplant patients are susceptible to infections due to their weakened immune response from prolonged immunosuppressive therapy (1). While preservation fluid (*PF*) plays a pivotal role in organ protection, it may concurrently act as a conducive medium for microbial growth. Early post-transplant infections and pathogens in *PF* have been linked in previous research (2–8). Furthermore, many studies recommend the use of antimicrobial or antifungal drugs as preemptive antibiotic therapy specifically targeting pathogens detected in *PF* (2, 9–13).

Donor-derived infections (DDIs) are a serious complication that can lead to significant graft loss, morbidity, and mortality (14). However, the risk of donor-transmitted infections varies among different pathogens. For instance, *coagulase-negative staphylococci* (CoNS) are relatively non-toxic gram-positive bacteria, presenting a lower transmission risk (4). Concurrently, prior studies have indicated that *Enterococcus faecium*, *Enterococcus faecalis*, and *Staphylococcus aureus* are associated with DDIs (4, 15). Gram-positive bacteria are frequently found in *PF* after renal transplantation, yet there are no well-established guidelines or consensus on their management. There is an urgent need for evidence regarding the risk stratification of gram-positive bacteria in *PF*.

Infections caused by multidrug-resistant organisms (MDROs) have emerged as a recent threat to solid organ transplantation (SOT) (16). Previous studies have indicated that multidrug-resistant-gram-positive bacteria (MDR-GP) accounted for three-quarters of MDROs on culture in solid organ transplantation donors (17). However, there is limited research on the impact of MDR-GP. Moreover, donors harboring MDR-GP are not typically considered unsuitable for transplantation. Therefore, it is imperative to investigate their role in post-transplant infections.

Preemptive antibiotic therapy in the perioperative period of kidney transplant varies widely (9, 18), particularly concerning gram-positive bacteria in *PF*. Commonly used agents include trimethoprim-sulfamethoxazole (TMP-SMX), first-and second-generation cephalosporins. However, due to intrinsic and acquired resistance mechanisms, these regimens do not reliably cover certain gram-positive bacteria (19, 20). Conventional antimicrobial drug regimens as a part of preemptive antibiotic therapy after kidney transplants have effective control of gram-negative bacteria (21). For some highly virulent gram-positive bacteria, incorporating anti-gram-positive antibiotic into preemptive antibiotic therapy is essential for effective infection management. However, scant research has investigated the impact of preemptive-anti-gram-positive antibiotic (P-antiGP) therapy against gram-positive bacteria in *PF*.

This study aimed to stratify the risk of gram-positive bacteria in *PF*. Furthermore, we investigated antibiotic resistance and its role in early post-kidney transplant infections. Lastly, we evaluated the efficacy of P-antiGP therapy.

## Materials and methods

We retrospectively analyzed 1,395 kidney transplant recipients in the First Affiliated Hospital of Sun Yat-sen University, China. Recipients without gram-positive bacteria in *PF* and those who underwent living donor kidney transplants were excluded. In addition, the clinical characteristics of donors and recipients, microbes in *PF*, infection events within 30 days post-transplantation, and preemptive antibiotic therapy were collected. Our study was approved by the research ethics committee of the First Affiliated Hospital of Sun Yat-sen University (approval number 2022439). All patient data were analyzed anonymously. Therefore, additional informed consent was waived. This study was conducted in accordance with the principles of the World Medical Association Declaration of Helsinki and the declaration of Istanbul.

### Microbial culture

Graft preservation fluid consisting of hyperosmotic citrate purine solution (S400, Shanghai, China) was used for graft perfusion during organ procurement and storage. Prior to the back-table kidney preparation, 10 mL of *PF* was extracted from the kidney storage bag. These samples were aseptically transferred to blood culture bottles. We noted any evidence of microbial growth. For microbial evaluations, the Bact/Alert 3D system (bioMérieux, Marcy l’Etoile, France) was employed to process *PF* samples. Antimicrobial Susceptibility Testing (AST) was performed in accordance with the manufacturer’s instructions using the VITEK^®^ 2 system (software version 8.01) with AST-N334, AST-N335, and AST-P639 cards for the corresponding bacterial groups, including *staphylococci*, *enterococci*, and *streptococci*.

### Definition and categorization

The definitions for infection events are detailed in a previous study (2). A bloodstream infection, including central and non-central line-associated bloodstream infection, was defined as recipients having positive microbial results in the bloodstream (22). A wound infection was denoted when symptoms such as pain, tenderness, localized swelling, erythema, or heat at the incision were observed. This diagnosis was later confirmed by detecting a positive pathogen result from an aseptically obtained sample of the wound (23). Criteria for a graft-site infection entail finding microbes in peri-kidney allograft fluid collections, excluding instances of potential bacterial colonization. The criteria for urinary tract infections, pneumonia, and infectious diarrhea adhere to the guidelines set by the Centers for Disease Control and Prevention/National Healthcare Safety Network (24–26). Multidrug resistance (MDR) refers to acquired resistance to at least one agent in three or more antimicrobial categories, while extensive drug resistance (XDR) is resistance to all agents except those in two antimicrobial categories (27). A diagnosis of possible donor-derived infections (P-DDIs) was made when there was a match in microbial species and antibiotic resistance between donor and recipient, with the latter manifesting relevant symptoms (28).

Recipients with any of the *E. faecium, S. aureus, and E. faecalis* in *PF* were categorized into the high-risk group, while those presenting with other gram-positive bacteria fell into the low-risk group. Preemptive antibiotic therapy is characterized as a targeted antibiotic or antifungal administration immediately post-transplant, aiming to counteract isolates from culture-positive *PF*, in the absence of overt clinical signs of active infection in the recipient (10). P-antiGP therapy refers to the addition of linezolid to the existing preemptive antibiotic therapy.

### Prophylactic measures and immunosuppressive protocols

Before December 31, 2018, all recipients were treated with cephalosporins as the routine perioperative antibacterial agent. After this date, recipients switched to carbapenem. Those at high-risk for fungal infections received Echinocandins like Caspofungin or Micafungin. Linezolid, as a P-antiGP, was administered intravenously to recipients potentially at risk of infections due to gram-positive bacteria. The immunosuppressive protocol began with basiliximab (20 mg on days 0 and 4, total 40 mg) or rabbit anti-thymocyte globulin (ATG; total dose 3–5 mg/kg, administered over a 3–5-day course), followed by decreasing steroid doses and a combination of mycophenolic acid with either tacrolimus or cyclosporin A. No recipients in this cohort underwent desensitization therapy due to elevated panel reactive antibody levels.

### Statistical analysis

Categorical data were presented as percentages and analyzed using the chi-square or Fisher’s exact test. Continuous variables, depending on their distribution, were either presented as mean with standard deviation (SD) or as median with interquartile range (IQR). They were assessed using the student’s *t*-test or Mann–Whitney U test. *p* < 0.05 was considered statistically significant. Univariate logistic regression was performed to identify potential risk factors for early post-transplant infection events. Variables with *p* < 0.1 in the univariate analysis were included in the multivariate logistic regression model. Odds ratios (ORs) with 95% confidence intervals (CIs) were reported. Kaplan–Meier analysis was used to assess death-censored graft survival, and comparisons between groups were performed using the log-rank test. Analyses were conducted using SPSS 26.0 (IBM, NY) and R software (version 4.3.1, R Foundation for Statistical Computing, Vienna, Austria).

## Results

### Characteristics of donors and recipients

**Figure 1:**
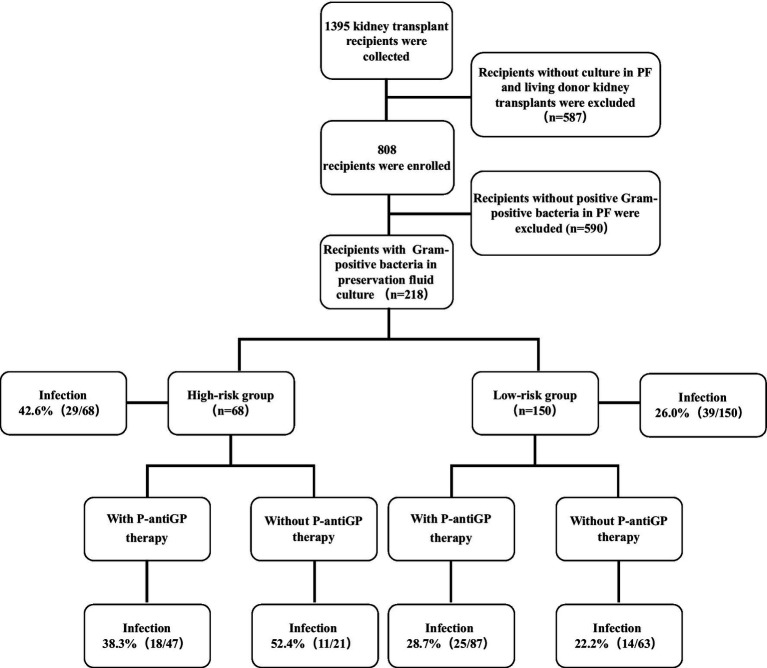
Relation between gram-positive bacteria in the *PF* as well as P-antiGP therapy and infection incidence rates. Recipients with any of the *E. faecium*, *E. faecalis,* and *S. aureus* in their *PF* regard as high-risk group. Recipients with other gram-positive bacteria in their *PF* regard as low-risk group.

The flow chart of this study is shown in Figure 1. The clinical characteristics of both donors and recipients are detailed in Table 1. Our analysis encompassed data from 218 kidney transplant recipients. The average age of these recipients was 37.6 years, with males accounting for 60.6%. Notably, 13.8% of the recipients demonstrated delayed graft function post-transplantation.

**Table 1:** Donor and recipient characteristics.

Variables		Group		
	Total (*N* = 218)	High-risk (*N* = 68)	Low-risk (*N* = 150)	*p*-value^a^
Recipients				
Age, years, mean ± SD	37.6 ± 18.3	39.7 ± 16.8	36.6 ± 18.9	0.227
Male, n (%)	132 (60.6)	38 (55.9)	94 (62.7)	0.342
Diabetes, n (%)	5 (2.3)	2 (2.9)	3 (2.0)	0.648
Induction therapy n (%)				0.668
ATG	143 (65.6)	46 (67.6)	97 (64.7)	
IL-2RA	75 (34.4)	22 (32.4)	53 (35.3)	
Dialysis type, n (%)				0.120
Hemodialysis	110 (50.5)	32 (47.1)	78 (52.0)	
Peritoneal dialysis	50 (22.9)	12 (17.6)	38 (25.3)	
None	58 (26.6)	24 (35.3)	34 (22.7)	
DGF, n (%)	30 (13.8)	12 (17.6)	18 (12.0)	0.262
Cephalosporin use (vs. carbapenem), n (%)	50 (22.9)	17 (25.0)	33 (22.0)	0.753
Donors				
Age, years, mean ± SD	31.0 ± 21.2	31.7 ± 21.3	30.7 ± 21.2	0.741
Male, n (%)	148 (68.8)	43 (64.2)	105 (70.9)	0.321
ICU stay, days, mean (IQR)	6.4 (5.4)	8.62 (8.1)	5.6 (3.9)	0.306
Cause of death, n (%)				0.366
Head trauma	90 (41.3)	26 (38.2)	64 (42.7)	
Cerebrovascular/stroke	64 (29.4)	21 (30.9)	43 (28.7)	
Anoxia	41 (18.8)	10 (14.7)	31 (20.7)	
CNS tumor	3 (1.4)	1 (1.5)	3 (2.0)	
Others	12 (5.5)	6 (8.8)	6 (4.0)	
Unknown	7 (3.2)	4 (5.9)	3 (2.0)	
Donor type, n (%)				0.242
DBD	172 (78.9)	47 (69.1)	125 (83.3)	
DCD	38 (17.4)	14 (20.6)	24 (16.0)	
Unknown	8 (3.7)	7 (10.3)	1 (0.7)	
Cold ischemia time in hours, mean (IQR)	10.5 (4.4)	11.6 (4.5)	10.0 (4.3)	0.018
Warm ischemia time in mins, mean (IQR)	2.8 (4.6)	4.7 (6.7)	2.0 (3.1)	0.003
Combined transplantation, n (%)	6 (5.7)	3 (7.9)	3 (4.4)	0.664

^a^Comparison between high-risk group and low-risk group. ATG, anti-thymocyte globulin; IL-2RA, Interleukin 2 receptor antibody; DBD, donation after brain death; DCD, donation after circulatory death; DGF, delayed graft function; SD, standard deviation; IQR, interquartile range; CNS, central nervous system.

A total of 218 kidney transplants were obtained from 144 deceased donors. The average age of the donors was 31.0 years and 78.9% of them were donated after brain death. Characteristics of both donors and recipients between the high-risk and low-risk groups showed no significant difference except for ischemia time. The high-risk group had a longer cold ischemia time of 11.6 h, compared to the 10.0 h in the low-risk group (*p* = 0.018). Additionally, the warm ischemia time in the high-risk group was significantly prolonged at 4.7 min, compared to the 2.0 min observed in the low-risk group (*p* = 0.003).

### Details of gram-positive bacteria in preservation fluid

**Figure 2:**
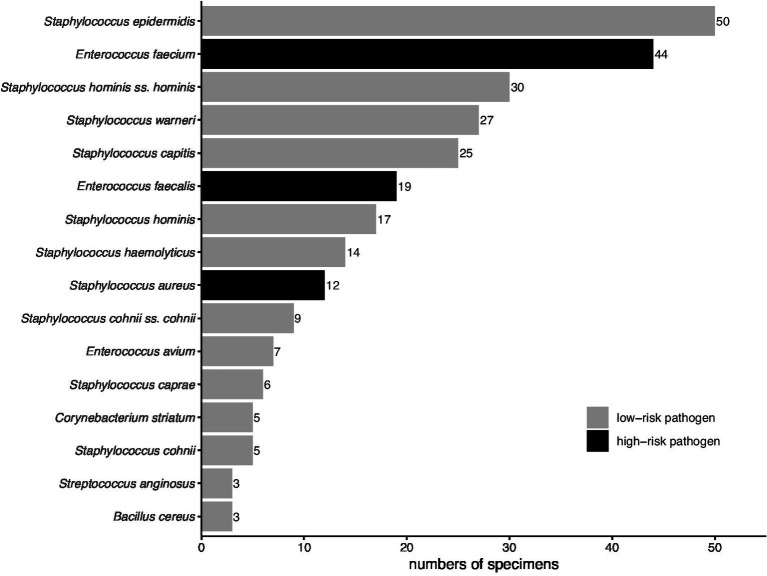
Distribution of gram-positive bacteria in Preservation fluid. Pathogens with a frequency of less than three are not shown.

Among the 218 kidney transplant recipients, 301 gram-positive bacterial isolates were identified in the *PF*. Isolation details of pathogens were as shown in Figure 2. The high-risk group accounted for 24.9% (75/301) of these isolates, with the remaining 75.1% (226/301) falling into the low-risk group. In the high-risk group, *E. faecium*, *E. faecalis*, and *S. aureus* accounted for 58.7% (44/75), 25.3% (19/75), and 16.0% (12/75) of the isolates, respectively. Meanwhile, CoNS represented a significant majority of the low-risk group, accounting for 88.9% (201/226) of its isolates.

### Infection incidence variation between risk groups

**Figure 3:**
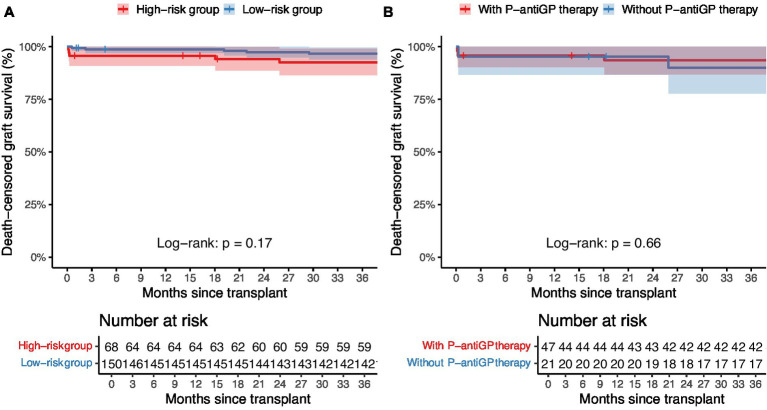
Death-censored graft survival stratified by pathogen risk and P-antiGP therapy. Kaplan–Meier survival curves comparing death-censored graft survival between **(A)** high-risk and low-risk pathogen groups, and **(B)** patients with or without P-antiGP therapy. Shaded areas represent 95% confidence intervals.

The current study further examined the association between distinct risk groups and post-transplantation infection events. 42.6% of the high-risk group (29/68) and 26.2% of the low-risk group (39/150) encountered at least one infection episode post-transplantation. Notably, the high-risk group exhibited a significantly heightened prevalence of overall infection (42.6% vs. 26.0%, *p* = 0.014), bloodstream infection (19.1% vs. 8.7%, *p* = 0.027), wound infection (7.4% vs. 0.7%, *p* = 0.012), and graft-site infection (23.5% vs. 6.7%, *p* < 0.001), compared to their low-risk group. Additionally, seven recipients (1.5%) were diagnosed with P-DDIs, all occurring in the high-risk group (*p* < 0.001) and caused by *E. faecium, E. faecalis, and S. aureus*, predominantly presenting as graft-site infections (Supplementary Table 5). However, no significant variation was observed in the incidence of pneumonia, urinary tract infection, and infectious diarrhea between the high-risk and low-risk groups (Table 2). We further assessed graft outcomes between pathogen risk groups. Although the high-risk group had a higher incidence of infections, including P-DDIs, death-censored graft survival was comparable between groups (log-rank *p* = 0.17) (Figure 3A). However, one recipient in the high-risk group underwent graft nephrectomy due to a severe *S. aureus* graft-site infection.

**Table 2:** Infection incidence variation between risk groups.

Infection events	Low-risk group (*n* = 150)		High-risk group (*n* = 68)		*P*-value
Pneumonia	13	8.7%	9	13.2%	0.300
Bloodstream infection	13	8.7%	13	19.1%	0.027
Wound infection	1	0.7%	5	7.4%	0.012
Graft-site infection	10	6.7%	16	23.5%	<0.001
Urinary tract infection	12	8.0%	4	5.9%	0.783
Infectious diarrhea	5	3.3%	6	8.8%	0.167
P-DDIs	0	0.0%	7	10.3%	<0.001
Overall infection	39	26.0%	29	42.6%	0.014

### Risk factors associated with early post-transplant infections

To identify variables associated with early post-transplant infections, univariate and multivariate logistic regression analyses were performed (Supplementary Table 4). Univariate analysis included the following variables: donor died of cerebrovascular accident, donor died of traumatic injuries, recipient gender, recipient age, hemodialysis, peritoneal dialysis, duration of dialysis, diabetes, preoperative hemoglobin, ATG induction versus basiliximab, cephalosporin versus carbapenem, delayed graft function, high-risk group, and *PF* pathogen antibiotic resistance. Most variables were not significantly associated with infection events. Variables with a *p* < 0.1 were delayed graft function (OR = 2.171, 95% CI: 0.982–4.765, *p* = 0.053), high-risk group (OR = 2.116, 95% CI: 1.156–3.877, *p* = 0.015), and PF pathogen antibiotic resistance (OR = 1.766, 95% CI: 0.987–3.210, *p* = 0.058), which were entered into the multivariate model.

In multivariate analysis, high-risk group remained marginally associated with infection events (adjusted OR = 1.855, 95% CI: 0.991–3.464, *p* = 0.052), whereas delayed graft function (adjusted OR = 1.899, 95% CI: 0.831–4.278, *p* = 0.122) and PF pathogen antibiotic resistance (adjusted OR = 1.609, 95% CI: 0.877–2.990, *p* = 0.127) did not retain statistical significance.

### Antibiotic resistance of gram-positive bacteria

Among the 218 recipients analyzed, 56.4% (123/218) were found with MDR-GP in their *PF*, while 12.4% (27/218) were detected extensively drug-resistant bacteria (XDR-GP), accounting for 15.2% (123/808) and 3.3% (27/808), respectively, of all PF-positive recipients. Notably, all pathogens isolated from P-DDI cases, including *E. faecium, E. faecalis, and S. aureus*, were multidrug-resistant (Supplementary Table 5). Among the high-risk recipients, 76.5% (52/68) presented with MDR-GP and 26.5% (18/68) with XDR-GP in the *PF*. For the low-risk group, 47.3% (71/150) had MDR-GP and 6% (9/150) had XDR-GP. Notably, the MDR group had higher rates of bloodstream infections (16.3% vs. 7.1%, *p* = 0.025) and overall infections (36.6% vs. 27.1%, *p* = 0.051) than the non-MDR group. The incidence of wound infection (11.1% vs. 1.6%, *p* = 0.026) and P-DDIs (11.1% vs. 2.1%, *p* = 0.014) in the XDR group were higher than those in the non-XDR group, as shown in Supplementary Table 1. In both high-risk and low-risk categories, recipients with drug-resistant pathogens in *PF* showed a higher incidence of infection events (Supplementary Tables 2, 3). There are 108 recipients in whom CoNS was the only pathogens detected in their *PF*. Of these, 62.0% (67/108) were identified as methicillin-resistant *coagulase-negative staphylococci* (MRSCoNS). Moreover, no significant differences were observed in infection events between the MRSCoNS and non-MRSCoNS groups (Table 3).

**Table 3:** The effect of methicillin-resistant c*oagulase-negative staphylococci* on early post-transplant infection.

Infection events	Non-MRSCoNS group (*n* = 41)		MRSCoNS group (*n* = 67)		*P*-value
Pneumonia	4	9.6%	4	6.0%	0.475
Bloodstream infection	2	4.9%	7	10.4%	0.478
Wound infection	0	0.0%	1	1.5%	1.000
Graft-site infection	1	2.4%	2	0.0%	1.000
Urinary tract infection	2	4.9%	7	10.4%	0.478
Infectious diarrhea	0	0.0%	3	4.5%	0.287
P-DDIs	0	0.0%	0	0.0%	–
Overall infection	7	17.1%	19	28.4%	0.183

### Effect of preemptive-anti-gram-positive antibiotic therapy

Of the 218 recipients, 58.0% (87/150) in the high-risk group and 69.1% (47/68) in the low-risk group added P-antiGP as preemptive therapy against gram-positive bacteria. Among the high-risk recipients, those administered with P-antiGP therapy experienced an infection rate of 38.3%, compared to 52.4% in those who did not. Additionally, the incidence of graft-site infection was 38.1% in recipients using P-antiGP therapy and 17.0% in those not using them. Notably, there was a significantly lower P-DDIs incidence of 4.3% in recipients administered with P-antiGP therapy, in contrast to 23.8% in those not given the antibiotic (*p* = 0.025) (Table 4). However, the low-risk group showed no significant difference in infection events regardless of P-antiGP therapy (Table 5). To further assess the safety of P-antiGP therapy, Kaplan–Meier analysis was performed and showed no significant difference in death-censored graft survival between the P-antiGP and non-P-antiGP groups (log-rank *p* = 0.66) (Figure 3B).

**Table 4:** Effect of P-antiGP therapy on infection control in the high-risk group.

Infection events	Without P-antiGP therapy (*n* = 21)		With P-antiGP therapy (*n* = 47)		*P*-value
Pneumonia	4	19.0%	5	10.6%	0.577
Bloodstream infection	6	28.6%	7	14.9%	0.321
Wound infection	2	9.5%	3	6.4%	0.641
Graft-site infection	8	38.1%	8	17.0%	0.113
Urinary tract infection	2	9.5%	2	4.3%	0.582
Infectious diarrhea	2	9.5%	4	8.5%	1.000
P-DDIs	5	23.8%	2	4.3%	0.025
Overall infection	11	52.4%	18	38.3%	0.278

**Table 5:** Effect of P-antiGP therapy on infection control in the low-risk group.

Infection events	Without P-antiGP therapy (*n* = 63)		With P-antiGP therapy (*n* = 87)		*P*-value
Pneumonia	5	7.9%	8	9.2%	0.787
Bloodstream infection	7	11.1%	6	6.9%	0.365
Wound infection	0	0.0%	1	1.1%	1.000
Graft-site infection	4	6.3%	6	6.9%	1.000
Urinary tract infection	4	6.3%	8	9.2%	0.526
Infectious diarrhea	0	0.0%	5	5.7%	0.074
P-DDIs	–	**–**	**–**	**–**	**–**
Overall infection	14	22.2%	25	28.7%	0.369

## Discussion

Infection continues to be a major concern for kidney transplant recipients (1). Although DDIs are infrequent, their implications can be severe (29). Current routine antibiotics inadequately addresses gram-positive bacteria in *PF* after kidney transplantation. Thus, it is imperative to assess the pathogenicities of different gram-positive bacteria in *PF* and to explore the influence of antibiotics on them. High-risk recipients with *E. faecium*, *S. aureus*, and *E. faecalis* in the *PF*, exhibited a significantly higher incidence of P-DDIs and other infections than the low-risk group in our investigation. Furthermore, our research noted a decrease in P-DDIs and infections when employing P-antiGP therapy for high-risk recipients. In contrast, no notable benefit was observed in the low-risk cohort. These findings may provide evidence for the use of P-antiGP therapy for certain patients presenting with gram-positive bacteria in *PF*.

In our study, 31.2% of the recipients with gram-positive bacteria in the *PF* were classified as high-risk. The factors that influence the presence of high-risk pathogens in *PF* have not been extensively explored in existing literature. We observed that the high-risk group experienced longer cold (11.6 h vs. 10.0 h) and warm (4.7 min vs. 2.0 min) ischemia times. A prior study on pancreas transplantation reported that recipients with positive *PF* culture exhibited longer cold ischemia time and warm ischemia time (3). Notably, prolonged cold and warm ischemia times may increase the prevalence of high-risk pathogens in *PF*. The increased duration potentially enhances the transmission risk of these pathogens from the donor. Consequently, we advocate for a reduction in ischemia time. Furthermore, our research showed that CoNS represented 90% of the pathogens in the low-risk group. In contrast, other study has found Enterococcus species to be the primary pathogen, with CoNS in second place (4). This shift might be attributed to the inclusion of amikacin in the *PF*. However, most studies consistently identified CoNS as the predominant pathogen (3, 6, 8, 10). Additionally, donor cause of death may influence the risk of donor-derived infections, which can potentially be anticipated through *PF* analysis. In our cohort, no significant difference in donor cause of death was observed across *PF* risk groups. Prior reports indicate that drowning donors can harbor waterborne or opportunistic organisms, such as *Aeromonas hydrophila* and *Legionella pneumophila* (30, 31) In our study, *Enterococcus faecium* was detected in two of four drowning donors and CoNS in the other two cases. Although different from the specific pathogens previously described, this phenomenon remains noteworthy.

Our study observed a significantly increased incidence of infection events in the high-risk group, predominantly associated with *E. faecium*, *E. faecalis*, and *S. aureus* in *PF*. In addition, multivariate analysis identified high-risk classification as a risk factor for post-transplant infections. Notably, these pathogens accounted for all P-DDIs in our cohort. The abundant virulence factors of *S. aureus*, combined with its ability to acquire resistance both within and between species, render it a particularly formidable and adaptive human pathogen (32). In our cohort, one recipient developed a severe P-DDI caused by *S. aureus* and subsequently underwent graft nephrectomy. *E. faecium* is commonly implicated in healthcare-associated infections, particularly in those who are immunocompromised (33). Notably, both *E. faecium* and *S. aureus* are categorized as ESKAPE pathogens, known for their contribution to hospital-acquired infections (34). Yu et al. (4) identified a heightened risk of P-DDIs in recipients exhibiting ESKAPE-positive *PF*. Previous research indicates that *E. faecalis* is linked to significant donor-derived infection incidents (15). Similarly, *E. faecalis*-associated P-DDI transmission events were observed in our cohort. Furthermore, *E. faecalis* is also a major opportunistic pathogen for critically ill or immuno-compromised patients (35). For the low-risk group, CoNS have gained increasing attention in recent years due to their frequent detection in sterile body fluids and infection sites (36). Previous studies have considered CoNS to be a skin-colonizing and contaminating bacterium (37). Consistent with our findings, a recent prospective study reported that most *PF* isolates were low-virulence organisms such as coagulase-negative *Staphylococcus*, and no *PF*-positive case led to a concordant infection (38). Given that CoNS has fewer virulence factors, recipients with it in *PF* are deemed low-risk group. Consequently, we advocate for the classification of CoNS into the low-risk pathogen category. Furthermore, it is imperative to monitor recipients harboring *E. faecium*, *E. faecalis*, and *S. aureus* in *PF* more diligently, given their correlation with elevated post-transplant infection risks.

It is imperative to maintain constant vigilance against MDR-GP in *PF* and to emphasize special scrutiny for XDR-GP. Recipients with MDR-GP in *PF* exhibited an elevated risk of post-transplant infections. Notably, all P-DDIs in our cohort were caused by MDR-GP, with XDR-GP significantly associated with DDIs. However, most donors with MDROs are not considered a contraindication to donation (39). The relationship between MDROs in *PF* and post-transplant recipient infections warrants increased scrutiny in this context. A study from the United States indicated that MDR-GP accounted for three-quarters of MDROs on culture in solid organ transplantation donors (17). Notably, despite the high prevalence of MDR-GP, literature on gram-positive bacteria in *PF* remains scarce. In our study, over half of the recipients identified MDR-GP in the *PF* samples. We observed an increased incidence of infection events post-kidney transplantation associated with MDR-GP in *PF*. Interestingly, the proportion of recipients detecting XDR-GP in the *PF* samples surpassed 10%. Meanwhile, we found that recipients with XDR-GP in *PF* have a higher P-DDIs incidence. It is crucial to closely monitor XDR-GP in *PF* to avert DDIs events. A pathogenetic analysis following liver transplantation revealed that MRSCoNS was the predominant bacterium at the post-operative surgical site (40). However, there is a lack of exploration of the risk of MRSCoNS in *PF*. In our investigation, there was no significant difference in the rate of infectious events between groups with MRSCoNS and those without, and none of them suffered from P-DDIs. This suggests that the likelihood of MRSCoNS transmission from the donor appears minimal, and its association with post-transplant infectious events is limited.

P-antiGP therapy may be essential for the high-risk group, but its benefits for those in the low-risk group appear constrained. Our research detected a diminished frequency of infection events in high-risk group when treated with P-antiGP therapy. Remarkably, the adoption of P-antiGP therapy also substantially decreased the incidence of P-DDIs in high-risk group. Consistent with our findings, most studies have demonstrated that preemptive antimicrobial therapy for recipients with pathogen-positive preservation fluid significantly reduces the risk of donor-derived infection transmission (2, 10, 41). Importantly, *E. faecium, S. aureus, and E. faecalis* have increasingly been recognized for their high virulence and potential to cause donor-derived infections, and most studies suggest that targeted preemptive therapy against these pathogens can effectively reduce postoperative infection rates (2, 10, 13, 41). However, a national investigation from France disclosed that only a third of kidney transplant recipients with *methicillin-susceptible Staphylococcus* in *PF* received antimicrobial prescribing (9). Given the current circumstances, we consider P-antiGP therapy may be appropriate for recipients with high-risk pathogens. Linezolid exhibits a strong efficacy as a P-antiGP. Consistent with our results, prior research also demonstrated its efficacy against enterococci and staphylococci in transplant recipients (42). Moreover, Linezolid typically requires minimal dosage adjustment in kidney transplant patients (43). Nonetheless, caution is warranted due to its myelosuppressive effects, which may potentially lead to thrombocytopenia (44). The use of vancomycin is discouraged due to its nephrotoxic effects. In addition, teicoplanin has high protein binding affinity, potential nephrotoxicity, and slow onset of action (45, 46). In our study, Kaplan–Meier analysis showed that P-antiGP therapy, including Linezolid, was not associated with adverse effects on long-term death-censored graft survival, supporting its potential safety in high-risk recipients. Conversely, the administration of P-antiGP therapy showed no impact on the infection event rates among the low-risk group. Similarly, Picola Brau et al. found that CoNS in *PF* rarely received preemptive antimicrobial therapy and were not associated with any donor-to-recipient transmission events, supporting the view that such low-virulence organisms may not warrant routine intervention (12). However, previous report indicated that 8.3% recipients received preemptive antibiotic therapy for methicillin-susceptible Staphylococcus in *PF* (9), we consider that this strategy might be less than optimal. In addition, previous studies suggested that combination use of antimicrobial drugs may increase the risk of intestinal dysbiosis (47). Consequently, for kidney transplant recipients with pathogens such as *E. faecium*, *E. faecalis*, or *S. aureus* present in their *PF*, we advocate the consideration of P-antiGP therapy. Yet, those with low-risk gram-positive bacteria might find such a regimen less advantageous.

There are limitations to our study. Firstly, it was a retrospective study. In addition, DDIs were not eligible for detection, so we used the concept of P-DDIs instead. Finally, the absence of donor pathogenicity data in our study precluded any related analysis.

In conclusion, we considered that *E. faecium*, *E. faecalis*, and *S. aureus* should be considered as high-risk gram-positive bacteria in *PF*. Recipients with any of these pathogens should also be considered as a high-risk group. The occurrence rate of antibiotic resistance in gram-positive bacteria within *PF* is notably high, leading to an increased incidence for infection events after kidney transplant. Meanwhile, despite the high occurrence of MRSCoNS in *PF*, their influence on infection rates seems to be negligible. Moreover, we advocate for the use of linezolid as P-antiGP therapy in high-risk group. Concurrently, P-antiGP therapy might not be advisable for recipients harboring alternate pathogens in *PF*.

## Data Availability

The raw data supporting the conclusions of this article will be made available by the authors, without undue reservation.
