# Supplementary material for: Preemptive antibiotic strategies for gram-positive bacteria in preservation fluid: a single-center experience
Source: Front Med (Lausanne). 2025 Sep 10;12:1665151. doi: 10.3389/fmed.2025.1665151 (PMC12457367; doi:10.3389/fmed.2025.1665151)
Supplement: Supplementary file 1 [file Table_1.docx]

| **Supplementary Table1** Impact of Drug Resistance on Infection Events | | | | | | | | | | | |
| --- | --- | --- | --- | --- | --- | --- | --- | --- | --- | --- | --- |
| Infection events | MDR (n=123) | | Non- MDR (n=85) | | P-value | XDR (n=27) | | Non- XD (n=191) | | P-value |  |
| Pneumonia | 12 | 9.8% | 10 | 11.8% | 0.852 | 3 | 11.1% | 19 | 9.9% | 0.741 |  |
| Bloodstream infection | 20 | 16.3% | 6 | 7.1% | 0.025 | 6 | 22.2% | 20 | 10.5% | 0.078 |  |
| Wound infection | 5 | 4.1% | 1 | 1.2% | 0.178 | 3 | 11.1% | 3 | 1.6% | 0.026 |  |
| Graft-site infection | 17 | 13.8% | 9 | 10.6% | 0.326 | 5 | 18.5% | 21 | 11.0% | 0.259 |  |
| Urinary tract infection | 8 | 6.5% | 8 | 9.4% | 0.590 | 2 | 7.4% | 14 | 7.3% | 1.000 |  |
| Infectious diarrhea | 9 | 7.3% | 2 | 2.4% | 0.119 | 2 | 7.4% | 9 | 4.7% | 0.631 |  |
| P-DDIs | 6 | 4.9% | 1 | 0.8% | 0.141 | 3 | 11.1% | 4 | 2.1% | 0.014 |  |
| Overall infection | 45 | 36.6% | 23 | 27.1% | 0.051 | 10 | 37.0% | 58 | 30.4% | 0.484 |  |

MDR, multidrug drug resistant; XDR-GP, extensively drug resistant

| **Supplementary Table2** Impact of Drug Resistance on Infection Events in High-risk Groups | | | | | | | | | |  |  |  |
| --- | --- | --- | --- | --- | --- | --- | --- | --- | --- | --- | --- | --- |
| Infection events | | MDR (n=52) | | Non- MDR(n=16) | | P-value | XDR (n=18) | | Non- XDR (n=50) | | | P-value |
| Pneumonia | | 7 | 13.5% | 2 | 12.5% | 1.000 | 3 | 16.7% | 6 | 12.0% | | 0.690 |
| Bloodstream infection | | 12 | 23.1% | 1 | 6.3% | 0.273 | 5 | 27.8% | 8 | 16.0% | | 0.306 |
| Wound infection | | 5 | 9.6% | 0 | 0.0% | 0.330 | 3 | 16.7% | 2 | 4.0% | | 0.111 |
| Graft-site infection | | 13 | 25.0% | 3 | 18.8% | 0.744 | 5 | 27.8% | 11 | 22.0% | | 0.259 |
| Urinary tract infection | | 2 | 3.8% | 2 | 12.5% | 0.233 | 1 | 5.6% | 3 | 6.0% | | 1.000 |
| Infectious diarrhea | | 6 | 1.9% | 0 | 0.0% | 0.323 | 2 | 11.1% | 4 | 8.0% | | 0.652 |
| P-DDIs | | 6 | 11.5% | 1 | 6.3% | 1.000 | 3 | 16.7% | 4 | 8.0% | | 0.371 |
| Overall infection | | 24 | 46.2% | 5 | 31.3% | 0.292 | 8 | 44.4% | 21 | 42.0% | | 0.857 |

MDR, multidrug drug resistant; XDR-GP, extensively drug resistant

| **Supplementary Table3** Impact of Drug Resistance on Infection Events in Low-risk Group | | | | | | | | | |  |  |  |
| --- | --- | --- | --- | --- | --- | --- | --- | --- | --- | --- | --- | --- |
| Infection events | | MDR (n=71) | | Non- MDR (n=79) | | P-value | XDR (n=9) | | Non- XDR (n=141) | | | P-value |
| Pneumonia | | 5 | 7.0% | 8 | 10.1% | 0.503 | 0 | 0% | 13 | 9.2% | | 1.000 |
| Bloodstream infection | | 8 | 11.3% | 5 | 6.3% | 0.283 | 1 | 11.1% | 12 | 8.5% | | 0.568 |
| Wound infection | | 0 | 0% | 1 | 1.3% | 1.000 | 0 | 0% | 1 | 0.7% | | 1.000 |
| Graft-site infection | | 4 | 5.6% | 6 | 7.6% | 0.749 | 0 | 0% | 10 | 7.1% | | 1.000 |
| Urinary tract infection | | 6 | 8.5% | 6 | 7.6% | 0.847 | 1 | 11.1% | 11 | 7.8% | | 0.538 |
| Infectious diarrhea | | 3 | 4.2% | 2 | 2.5% | 0.668 | 0 | 0% | 5 | 3.5% | | 1.000 |
| P-DDIs | | - | - | - | - | - | - | - | - | - | | - |
| Overall infection | | 21 | 30.0% | 18 | 22.8% | 0.344 | 2 | 22.2% | 37 | 26.2% | | 1.000 |

MDR, multidrug drug resistant; XDR-GP, extensively drug resistant

| **Supplementary Table4** Univariate and Multivariate Logistic Regression Analysis of Risk Factors for Early Post-Transplant Infection Events | | | | |
| --- | --- | --- | --- | --- |
| Variables | Unadjusted OR (95% CI) | P-value | Adjusted OR (95% CI) | P-value |
| Donor died of cerebrovascular accident | 1.501(0.614, 3.879) | 0.383 | - | - |
| Donor died of traumatic injuries | 1.778(0.774, 4.388) | 0.190 | - | - |
| Recipient gender | 0.901(0.503,1.623) | 0.725 | - | - |
| Recipient age | 1.011(0.995,1.028) | 0.182 | - | - |
| Hemodialysis | 1.155(0.651,2.057) | 0.622 | - | - |
| Peritoneal dialysis | 0.93(0.458,1.823) | 0.836 | - | - |
| Duration of dialysis | 1.055(0.916,1.210) | 0.446 | - | - |
| diabetes | 0.996(0.981,1.010) | 0.567 | - | - |
| Preoperative hemoglobin | 0.894(0.412,1.845) | 0.768 | - | - |
| ATG induction (vs. basiliximab) | 1.537(0.832,2.916) | 0.178 | - | - |
| Cephalosporin use (vs. Carbapenem), n (%) | 1.667(0.856,3.206) | 0.128 | - | - |
| Delayed graft function | 2.171(0.982,4.765) | 0.053 | 1.899(0.831,4,278) | 0.122 |
| High-risk group | 2.116(1.156,3.877) | 0.015 | 1.855(0.991,3.464) | 0.052 |
| PF pathogen antibiotic resistance | 1.766(0.987,3.21) | 0.058 | 1.609(0.877,2.990) | 0.127 |

Univariate and multivariate logistic regression analysis of risk factors for early post-transplant infection events. Variables with P < 0.1 in univariate analysis were included in the multivariate logistic regression model. Odds ratios (OR) are presented with 95% confidence intervals (CI). ATG, rabbit anti-thymocyte globulin.

| **Supplementary Table5** Clinical Profiles of Seven Recipients Diagnosed with Possible Donor-Derived Infections | | | | | |
| --- | --- | --- | --- | --- | --- |
| Case No. | Pathogens of P-DDIs | Presence of ESBL-producing or Multidrug-resistant Gram-negative Co-infection | Possible Donor-Derived Infections Infection Manifestation | Gram-positive Resistance Profile | Post-transplant Outcome |
| 1 | *Enterococcus faecium* | No | Graft-site infection, bloodstream infection | Ampicillin-R, Gentamicin-R, Erythromycin-R, Levofloxacin-R | Survival |
| 2 | *Staphylococcus aureus* | No | Graft-site infection, bloodstream infection, wound infection, urinary tract infection | Penicillin-R, Erythromycin-R, Clindamycin-R, Levofloxacin-R | Graft Nephrectomy |
| 3 | *Enterococcus faecium* | No | Graft-site infection, bloodstream infection, wound infection | Ampicillin-R, Streptomycin-R, Erythromycin-R, Clindamycin-R, Levofloxacin-R | Survival |
| 4 | *Enterococcus faecium* | No | Graft-site infection | Erythromycin-R, Clindamycin-R, Ceftriaxone-R, Ofloxacin-R | Survival |
| 5 | *Enterococcus faecalis* | No | Graft-site infection | Ampicillin-R, Erythromycin-R, Levofloxacin-R | Survival |
| 6 | *Enterococcus faecium, Klebsiella pneumoniae* | *Klebsiella pneumoniae*; Amikacin-R, Ertapenem-R, Ampicillin/Sulbactam-R, Polymyxin B-R, Minocycline-R, Ceftazidime-R, Piperacillin-R | Graft-site infection, bloodstream infection, wound infection, urinary tract infection | Ampicillin-R, Erythromycin-R, Clindamycin-R, Levofloxacin-R | Survival |
| 7 | *Enterococcus faecium*, *Candida albicans*(non-resistant) | No | Graft-site infection | Ampicillin-R, Erythromycin-R, Clindamycin-R, Levofloxacin-R | Survival |

Abbreviations: P-DDIs, possible donor-derived infections; ESBL, extended-spectrum β-lactamase; MDR, multidrug-resistant; R, resistant; GP, Gram-positive; PF, preservation fluid; *E. faecium, Enterococcus faecium*; *E. faecalis*, *Enterococcus faecalis*; *S. aureus*, *Staphylococcus aureus*; *K. pneumoniae*, *Klebsiella pneumoniae*.
